# Elevated markers of gut leakage and inflammasome activation in COVID‐19 patients with cardiac involvement

**DOI:** 10.1111/joim.13178

**Published:** 2020-10-08

**Authors:** H. Hoel, L. Heggelund, D. H. Reikvam, B. Stiksrud, T. Ueland, A. E. Michelsen, K. Otterdal, K. E. Muller, A. Lind, F. Muller, S. Dudman, P. Aukrust, A. M. Dyrhol‐Riise, J. C. Holter, M. Trøseid

**Affiliations:** ^1^ Research Institute of Internal Medicine Oslo University Hospital Oslo Norway; ^2^ Lovisenberg Diaconal Hospital Oslo Norway; ^3^ Institute of Clinical Medicine University of Oslo Oslo Norway; ^4^ Department of Clinical Science Faculty of Medicine University of Bergen Bergen Norway; ^5^ Department of Internal Medicine Drammen Hospital Vestre Viken Hospital Trust Drammen Norway; ^6^ Department of Infectious Diseases Oslo University Hospital Oslo Norway; ^7^ Faculty of Health Sciences K.G. Jebsen TREC University of Tromsø Tromsø Norway; ^8^ Department of Microbiology Oslo University Hospital Oslo Norway; ^9^ Section of Clinical Immunology and Infectious Diseases Oslo University Hospital Oslo Norway

**Keywords:** CCR9, COVID‐19, gut, heart, inflammasome, LBP

## Abstract

**Background:**

A high proportion of COVID‐19 patients have cardiac involvement, even those without known cardiac disease. Downregulation of angiotensin converting enzyme 2 (ACE2), a receptor for SARS‐CoV‐2 and the renin‐angiotensin system, as well as inflammatory mechanisms have been suggested to play a role. ACE2 is abundant in the gut and associated with gut microbiota composition. We hypothesized that gut leakage of microbial products, and subsequent inflammasome activation could contribute to cardiac involvement in COVID‐19 patients.

**Methods:**

Plasma levels of a gut leakage marker (LPS‐binding protein, LBP), a marker of enterocyte damage (intestinal fatty acid binding protein, IFABP), a gut homing marker (CCL25, ligand for chemokine receptor CCR9) and markers of inflammasome activation (IL‐1β, IL‐18 and their regulatory proteins) were measured at three time points (day 1, 3–5 and 7–10) in 39 hospitalized COVID‐19 patients and related to cardiac involvement.

**Results:**

Compared to controls, COVID‐19 patients had elevated plasma levels of LBP and CCL25 but not IFABP, suggesting impaired gut barrier function and accentuated gut homing of T cells without excessive enterocyte damage. Levels of LBP were twice as high at baseline in patients with elevated cardiac markers compared with those without and remained elevated during hospitalization. Also, markers of inflammasome activation were moderately elevated in patients with cardiac involvement. LBP was associated with higher NT‐pro‐BNP levels, whereas IL‐18, IL‐18BP and IL‐1Ra were associated with higher troponin levels.

**Conclusion:**

Patients with cardiac involvement had elevated markers of gut leakage and inflammasome activation, suggestive of a potential gut‐heart axis in COVID‐19.

## Graphical abstract

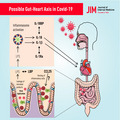

## Introduction

A substantial proportion of hospitalized COVID‐19 patients have cardiac involvement [1, 2]. Previous cardiovascular disease (CVD) and risk factors for CVD such as obesity seem to be major risk factors for developing severe COVID‐19 [1, 2, 3, 4]. However, a high proportion of COVID‐19 patients have cardiac involvement without previous CVD [2]. Cardiac involvement has also emerged as a significant and life‐threatening complication in COVID‐19 patients, ranging from myocardial infarction (MI) and myocarditis to pulmonary hypertension with cardiac stress [5, 6, 7, 8, 9].

The mechanisms underlying this cardiac involvement are not clear. Downregulation of the anti‐inflammatory and cardioprotective angiotensin (AT)‐1‐7 pathway secondary to downregulation of angiotensin converting enzyme ACE2, the SARS‐CoV‐2 receptor, direct infection of the myocardium through ACE2 expressing cardiac cells and hyperinflammation have been suggested to be of importance [10, 11, 12, 13]. ACE2 is ubiquitously expressed in several organs, and in addition to pulmonary, cardiac and renal tissues, ACE2 is also expressed in the gut, where ACE2 expression in enterocytes may serve as sites for SARS‐CoV‐2 entrance and predispose to enteric infection [14].

Data suggest that severe SARS‐CoV‐2 infection could lead to degeneration of the gut‐blood barrier leading to systemic spread of bacteria and leakage of microbial products, possibly affecting the host’s response to COVID‐19 infection and contribute to disease severity [15, 16, 17, 18, 19, 20]. A large GWAS study identified mutations in the chemokine receptor CCR9 as a major risk factor for developing severe COVID‐19 [21]. CCR9 is differentially expressed on T cells and regulate homing of T cells to the mucosa of the small intestines and the colon via interaction with the CCR9 ligand CCL25 [22, 23]. This underscores a potential role of gut mucosal function and inflammation in the pathogenesis of COVID‐19‐related disease.

An accumulating amount of evidence has shown that the gut microbiota composition and the gut‐blood barrier are altered in various forms of CVD [24]. Nod‐like Receptor Protein (NLRP) 3 inflammasome activation seems to play a major role in the pathogenesis of CVD, mainly through enhanced release of the inflammatory cytokines interleukin (IL)‐1β and IL‐18 [25, 26]. NLRP3 inflammasome activation has also been suggested as an important link between altered gut microbiota composition, impaired gut barrier and systemic inflammation [27], partly through priming of NLRP3 inflammasome through interaction between lipopolysaccharide (LPS) and toll‐like receptor (TLR)4 [28]. The NLRP3 inflammasome has also been proposed to play a role in the pathogenesis COVID‐19, but data on the role of these inflammasomes in the gut‐heart axis in COVID‐19 patients are scarce or lacking [29].

Herein, we measured plasma levels of LPS‐binding protein (LBP) and intestinal fatty acid binding protein (IFABP) as markers of disturbed gut barrier [30], CCL25 as a marker of T cell homing in the gut, and IL‐1 and IL‐18 as well as their regulatory proteins as the major products of NLRP3 inflammasome activation. We hypothesized that gut leakage mechanisms involving LPS‐ and CCL25‐driven intestinal inflammation through NLRP3 activation could contribute to cardiac involvement among hospitalized COVID‐19 patients.

## Methods

### Study population

Hospitalized adult patients (≥18 years old) with confirmed positive SARS‐CoV‐2 PCR test targeting the E‐gene on oro‐ or nasopharyngeal specimens, were consecutively recruited from Oslo University Hospital Ullevål and Drammen Hospital, Vestre Viken Hospital Trust between March 6 and April 14 to a clinical cohort study (Norwegian SARS‐CoV‐2 study; ClinicalTrials.gov number NCT04381819). Clinical information and laboratory samples were collected at the earliest time point after hospitalization. Peripheral blood was collected at day of inclusion (within 48 h of admission), day 3–5 and day 7–10 after hospitalization. Using a modified version of the International Severe Acute Respiratory and emerging Infection Consortium (ISARIC)/World Health Organization Clinical Characterization Protocol, clinical and routine data were abstracted from electronic medical records into the ISARIC (isaric.tghn.org) REDCap database (Research Electronic Data Capture, Vanderbilt University, USA, hosted by University of Oxford, UK).

For reference, the actual markers were also analysed in plasma from 16 healthy controls recruited at the Research Institute of Internal Medicine, OUH, based on disease history and normal laboratory tests (Table 1).

**Table 1:** Baseline characteristics of COVID‐19 patients and controls

	Controls	All patients	Cardiac involvement	
	(*n* = 16)	(*n* = 39)	No (*n* = 17)	Yes (*n* = 22)
Women, *n* (%)	7 (44)	10 (26)	6 (35)	4 (18)
Age, years	66 ± 7	61 ± 15	58 ± 13	63 ± 16
Time from symptoms, days	–	9.6 ± 3.7	9.7 ± 4.2	9.6 ± 3.2
Caucasian, *n* (%)	16 (100)	27 (69)	10 (59)	17 (77)
Current smoker, *n* (%)	3 (19)	8 (21)	1 (6)	7 (32)
P/F ratio		42.4 ± 15.3	47.0 ± 17.1	37.6 ± 12.2
Comorbidities				
Cardiovascular, *n* (%)	0 (0)	9 (23)	2 (12)	7 (32)
Pulmonary, *n* (%)	0 (0)	1 (2.6)	0 (0)	1 (4.5)
Asthma, *n* (%)	0 (0)	8 (21)	4 (24)	4 (18)
Renal, *n* (%)	0 (0)	4 (10)	0 (0)	4 (18)
Liver, *n* (%)	0 (0)	0 (0)	0 (0)	0 (0)
Neurological, *n* (%)	0 (0)	1 (3)	0 (0)	1 (5)
Cancer, *n* (%)	0 (0)	1 (3)	1 (6)	0 (0)
Hematological, *n* (%)	0 (0)	1 (3)	1 (6)	0 (0)
Obesity, *n* (%)	0 (0)	5 (13)	2 (12)	3 (14)
Diabetes, *n* (%)	0 (0)	3 (8)	1 (6)	2 (9)
Rheumatic, *n* (%)	0 (0)	4 (10)	1 (5)	3 (14)
Biochemistry				
Haemoglobin, g/dL	14.5 ± 0.9	13.3 ± 1.7**	12.7 ± 1.6	13.7 ± 1.7
Leucocytes, ×10^9^ L^−1^	5.6 ± 1.2	6.6 ± 3.2	5.3 ± 2.0	7.7 ± 3.6*
Lymphocytes, ×10^9^ L^−1^	1.7 ± 0.7	1.1 ± 0.5**	1.2 ± 0.5	1.0 ± 0.4
Monocytes, ×10^9^ L^−1^	0.5 ± 0.2	0.4 ± 0.2	0.5 ± 0.2	0.4 ± 0.2
Neutrophils, ×10^9^ L^−1^	3.2 ± 0.7	5.1 ± 3.2*	3.5 ± 1.9	6.3 ± 3.6*
Platelets, ×10^9^ L^−1^	254 ± 70	202 ± 59**	212 ± 52*	194 ± 66
CRP mg/L	1.6 [0.8, 4]	59 [31, 132]***	31 [15, 75]	109 [47, 179]**
ALT, U L^−1^	29 ± 13	43 ± 41	58 ± 55	32 ± 19
AST, U L^−1^	32 ± 9	49 ± 38	58 ± 47	36 ± 10

Continuous data are given as mean ± standard deviation. *P < 0.05, **P < 0.01 versus controls or COVID‐19 patients without cardiac involvement, respectively.

### Ethical considerations

Informed consents were obtained from all participants. The study was approved by the South‐Eastern Norway Regional Health Authority (reference number: 106624).

### Study outcome definitions

The main outcome was cardiovascular (CV) endpoint defined by cardiac markers above reference values at any time during hospitalization: N‐terminal pro‐B‐type natriuretic peptide (NT‐proBNP) reflecting cardiac wall stress (women: <50 years (y) 170–299 ng L^−1^; 50–69 y 300–759 ng L^−1^; ≥70 y ≥ 760 ng L^−1^, men: <50 y 85–249 ng L^−1^; 50–69 y 250–499 ng L^−1^; ≥70 y ≥ 500 ng L^−1^) and/or cardiac troponins reflecting myocardial damage: troponin T (TnT) (≥14 ng mL^−1^), or TnI (women ≥ 15 ng mL^−1^, men ≥ 30 ng mL^−1^). Cut‐off references as provided by local laboratories based on product information from Roche (NT‐proBNP and TnT) and Abbot (TnI).

### Sample processing

Peripheral blood was collected into 4 mL Vacuette^®^ (Greiner bio‐one International) with EDTA as anti‐coagulant. Samples were immediately stored on ice, and within 30 min plasma was isolated by centrifugation at 2000 ***g*** for 20 min at 4°C to obtain platelet poor plasma. Plasma were immediately stored at −80°C in aliquots until analysis.

### Markers of gut leakage and inflammasome activation

Plasma levels of IFABP, CCL25, IL‐18, IL‐18 binding protein (IL‐18BP) and LBP were measured in duplicate by enzyme immunoassays (EIA) using commercially available antibodies (R&D Systems, Minneapolis, MN, USA) in a 384 format using a combination of a SELMA (Jena, Germany) pipetting robot and a BioTek (Winooski, VT, USA) dispenser/washer. Absorption was read at 450 nm with wavelength correction set to 540 nm using an EIA plate reader (Bio‐Rad, Hercules, CA, USA). Plasma levels of IL‐1β and IL‐1Receptor antagonist (IL‐1Ra) were analysed using a multiplex cytokine assay (Bio‐Plex Human Cytokine 27‐Plex Panel; Bio‐Rad Laboratories Inc., Hercules, CA, USA). The samples were analysed on a Multiplex Analyzer (Bio‐Rad Laboratories) according to the manufacturer’s instructions.

### Statistical analysis

**Fig. 1:**
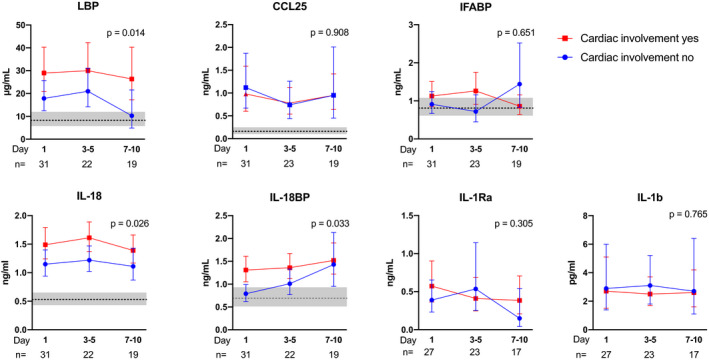
Circulating levels of gut and inflammasome markers in relation to cardiac involvement. Temporal course of gut‐related (upper panel) and inflammasome related (lower panel) markers during COVID‐19 infection according to cardiac involvement. Data are presented as back‐transformed estimated marginal means with 95% confidence intervals from the general linear model procedure (see Statistical methods) and the *P*‐value for the group effect according to cardiac involvement is given on the graphs. The grey area represents the estimated marginal mean (line) and 95% confidence interval (grey area) of healthy controls (*n* = 16).

Patient characteristics were compared using Student’s *t*‐test and Mann–Whitney *U* test or chi‐square for continuous and categorical variables, respectively (Table 1). The temporal profiles of IFABP, CCL25, LBP, IL‐18, IL18BP and IL‐1Ra, were analysed by a generalized linear mixed model. Markers were categorized as day 1 (i.e. within 48 h of admission), day 3–5 and day 7–10 giving three time categories (Figure 1) and were log transformed. Marker was used as dependent, outcome measure and time as fixed factors, and patient number as random factor.

We further used linear mixed models to model the association between inflammasome markers (outcome) and gut‐related biomarkers included individually in separate models, with time treated as a factor variable. A random intercept by subject was used to control for repeated measures, with each subject having between 1 and 3 measured follow‐up periods. Skewed data were log transformed and standardized.

Correlation analyses between markers of gut leakage and inflammasome activation were performed at individual time points (Spearman). The predictive value of LBP and inflammasome markers for troponin and NT‐proBNP levels was examined by logistic regression. LBP and inflammasome markers were log transformed and normalized, and levels of troponin and NT‐proBNP dichotomized according to gender and age‐related cut‐offs. *P*‐values are two‐sided and considered significant when <0.05. SPSS release 26.0.0.1 (IBM, Chicago, Illinois, USA) was used for statistical analysis.

## Results

### Demographics

Table 1 shows demographics and clinical characteristics of the 39 COVID‐19 patients and 16 healthy controls. COVID‐19 patients had a mean age of 61 years, 74% were men, 21% were current smokers, and 23% had previous CVD. Controls were slightly older with a mean age of 66 years, 56% were men, and 19% were current smokers. In COVID‐19 patients, the mean time from onset of symptoms to admission was 9.6 days.

In total, 56% of patients had elevated levels of cardiac markers above gender and age‐related cut‐offs as defined in Methods. No significant differences in age, time from symptoms to admission or major comorbidities including previous CVD were detected in relation to cardiac involvement.

Numerically a larger proportion of patients with cardiac involvement were current smokers, but the difference was not significant.

### COVID‐19 patients have elevated levels of LBP, CCL25 and markers of inflammasome activation

As shown in Figure 1, COVID‐19 patients had elevated plasma levels of LBP compared to controls at baseline (*P* < 0.001), suggesting impaired gut barrier function and endotoxin activity, while plasma levels of IFABP, a marker of enterocyte damage, were not elevated in COVID‐19 patients. Also plasma levels of CCL25 were elevated in COVID‐19 patients (*P* < 0.001), suggestive of a higher potential for influx of gut homing of T cells to the chemokine receptor CCR9. However, we found no correlation between lymphocyte count and CCL25 levels. Plasma levels of IL‐18 (*P* < 0.001) and IL‐18BP (*P* < 0.01), were also elevated in COVID‐19 patients compared to controls at baseline, suggesting increased inflammasome activation (Fig. 1). Plasma levels of IL‐1β and IL‐1Ra from COVID‐19 patients were measured by a multiplex cytokine assay, and not analysed in the controls. However, the levels were higher in COVID‐19 patients than that found in a healthy control group previously published by Hennø et al. [31] as reported for this COVID‐19 cohort (submitted paper). Time from symptoms´ onset did not differ between those with and without cardiac involvement and did not correlate with the analysed biomarkers or levels of NT‐proBNP or troponin.

### LPS‐driven inflammation and inflammasome activity in relation to cardiac involvement in COVID‐19 patients

When comparing COVID‐19 patients with elevated cardiac markers to those without, LBP levels were twice as high at day 1 in patients with cardiac involvement and remained higher at three time points during the hospital stay (Fig. 1). In contrast, CCL25 and IFABP, also related to gut inflammation and leakage, were not associated with cardiac involvement. Among markers of inflammasome activity, IL‐18 and IL‐18BP, but not IL‐1β and IL‐Ra were elevated throughout the hospital stay in patients with cardiac involvement (Fig. 1).

A large proportion of the COVID‐19 patients had previous history of CVD, but among patients with elevated cardiac markers, only IL‐18BP levels were elevated in patients with previous CVD as compared with those without (*P* = 0.045), whereas levels IL‐18 and LBP were not. There were no significant differences between smokers and non‐smokers in levels of LBP, IL‐18 and IL‐18BP. Furthermore, there was no difference in respiratory failure as measured by PaO_2_/FiO_2_ ratio between those with and without cardiac involvement (Table 1).

Finally, patients with cardiac involvement had higher levels of CRP, neutrophil counts and neutrophil:lymphocyte ratio. CRP levels correlated with LBP (rho = 0.63, *P* < 0.001), but not with markers of inflammasome activation.

### Markers of inflammasome activation are related to troponin levels and LBP to NT‐pro‐BNP

**Fig. 2:**
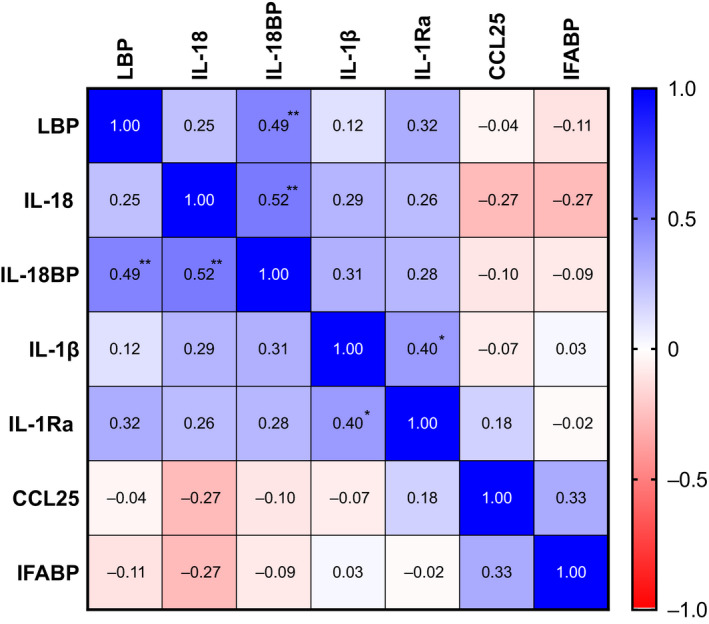
Correlation matrix at baseline. Correlation matrix at baseline with rho values and *P < 0.05, **P < 0.01, ***P> 0.001.

Correlations between biomarkers at baseline are presented as a correlation matrix in Fig.2, revealing positive correlations between IL‐18BP and LBP. To further investigate potential associations over time, we applied mixed models with markers of inflammasome activation as dependent variables and LBP and time as covariates. As shown in Table 2, LBP was significantly associated over time with IL‐18 and IL‐1Ra, supporting our overall hypothesis of LPS‐driven priming of the NLRP3 inflammasome.

**Table 2:** Association between markers of gut involvement and inflammasome activation over time

	IL‐18	IL‐18BP	IL‐1β	IL‐1Ra
LBP	**0.29** ** **(0.11, 0.47)**	0.18 (−0.07, 0.42)	−0.04 (−0.37, 0.29)	**0.34** * **(0.02, 0.66)**
CCL25	0.01 (−0.10, 0.12)	−0.08 (−0.30, 0.13)	0.06 (−0.20, 0.31)	**0.27** * **(0.03, 0.52)**
IFABP	0.07 (−0.17, 0.02)	0.02 (−0.19, 0.23)	−0.01 (−0.24, 0.24)	0.13 (−0.11, 0.37)

Linear mixed model with markers of inflammasome activation as dependent, time as fixed effect, and biomarker as covariates (LBP, CCL25 or IFABP), and subject as random effect. Data are given as parameter estimates (95%CI) and

* *P* < 0.05,

** *P* < 0.01.

Significant values are in bold.

As markers of inflammasome activation and gut barrier impairment could be related to different aspects of cardiac involvement, we analysed these markers in relation to elevated levels of troponin and NT‐pro‐BNP in logistic regression analyses. Whereas IL‐18, IL‐18BP and IL‐1Ra all associated with elevated troponin levels, but not with NT‐pro‐BNP, the opposite pattern was observed for LBP, which associated with elevated NT‐pro‐BNP but not with troponin levels (Table 3). Of note, LBP was not correlated with respiratory failure (PaO_2_/FiO_2_ ratio), suggesting that the association to NT‐pro‐BNP is not exclusively driven by increased pulmonary affection.

**Table 3:** Gut leakage and inflammasome activation in association with elevated cardiac markers

	LBP	IL18	IL18BP	IL‐1β	IL‐1Ra
Elevated Troponin	1.23 (0.74, 2.06)	**3.57 (1.70, 7.47)**	**4.68 (1.94, 11.29)**	1.53 (0.91, 2.59)	**2.03 (1.15, 3.58)**
	*P* = 0.431	***P* = 0.001**	***P* = 0.001**	*P* = 0.111	***P* = 0.015**
Elevated Nt‐proBNP	**2.62 (1.34, 5.12)**	1.40 (0.78, 2.53)	1.47 (0.85, 2.56)	1.23 (0.74, 2.04)	1.42 (0.84, 2.41)
	***P* = 0.005**	*P* = 0.265	*P* = 0.168	*P* = 0.428	*P* = 0.193

Logistic regression analyses with elevated levels of troponin and NT‐proBNP as outcome measures, dichotomized according to gender and age‐related cut‐offs given in Methods. Data are given as odds ratios (95% CI), with significant associations in bold.

## Discussion

In the present study, we hypothesized that gut leakage of microbial products, and subsequent inflammasome activation could contribute to cardiac involvement in COVID‐19 patients. Our findings can be summarized as follows: (i) Compared to controls, COVID‐19 patients had elevated plasma levels of LBP and CCL25, suggesting impaired gut barrier function and accentuated gut homing of T cells, (ii) baseline levels of LBP were twice as high in patients with cardiac involvement compared to those without and remained elevated during the hospital stay, (iii) IL‐18 and IL‐1Ra correlated with LBP over time and were significantly elevated in patients with current cardiac involvement and (iv) whereas inflammasome markers (IL‐18, IL‐18BP and IL‐1Ra) were associated with elevated troponin levels, LBP associated with elevated NT‐pro‐BNP.

Several studies, including work from our own group, have over the last years consistently reported gut microbiota alterations in cohorts of heart failure and coronary artery disease, with common findings being reduction in microbes with capacity for producing butyrate, which is vital for the gut mucosal function [24]. A dysfunctional gut mucosal barrier will facilitate passive leakage of microbial products, such as LPS to the circulation, which again could contribute to systemic inflammation at least partly through inflammasome activation. Moreover, in line with the present study, we recently reported that increased plasma levels of LBP predicted cardiovascular events in a high‐risk population of elderly men [32].

Gut impairment could trigger inflammation in several ways, among others by LPS‐priming of the NLRP3 inflammasome, which has been shown to play a central role in CVD [33, 34, 35] as well as in the pathogenesis of COVID‐19 [36]. Furthermore, the chemokine receptor CCR9 that is expressed by the intestinal epithelial cells, was recently identified in a large GWAS study as a potential risk factor for developing severe COVID‐19 diseases [21], underscoring a potential role of the gut in the pathogenesis of COVID‐19‐related disease. Of note, levels of LBP and the CCR9 ligand CCL25 were elevated in COVID‐19 patients, and it is tempting to hypothesize that LPS and CCL25 could contribute to gut mucosal impairment through promoting intestinal inflammation [37]. As COVID‐19 patients are often characterized by lymphopenia, we also hypothesized that this could partly be explained by gut homing of lymphocytes. However, we found no association between CCL25 levels and lymphocyte count.

Whereas levels of IL‐18 and its binding protein were associated with cardiac involvement in COVID‐19 patients, this was not seen for the other major inflammatory NLRP3 inflammasome product, IL‐1β. Nevertheless, in addition to IL‐18 and IL‐18BP, also IL‐1Ra associated with elevated troponin levels. The reason for this pattern is at present not clear but could be related to methodological issues. It is well known that an accurate measurement of IL‐1β in circulation could be difficult, and we cannot exclude that IL‐1β could be of importance for cardiac involvement in the microenvironment.

We recently reported that elevated circulating markers of inflammasome activation, including IL‐1Ra but not IL‐1β predicted first‐time MI in HIV [38], another viral infection where inflammasome activation is probably involved in disease pathogenesis through pyroptosis [39]. Strategies to inhibit IL‐1 activation by the monoclonal antibody canakinumab was recently shown to reduce the risk of re‐infarction in the CANTOS trial [25]. Retrospective cohort studies with IL‐1 inhibition with the short‐acting IL‐1 receptor antagonist anakinra in COVID‐19 patients have shown promising results [40] and is now being tested in several controlled trials, such as the CAN‐COVID study [29, 41, 42, 43].

Whereas markers of inflammasome activation were associated with elevated troponin levels reflecting myocardial damage, LBP levels associated with NT‐pro‐BNP, but not with troponin levels. Whether the remarkably high LBP levels in patients with elevated cardiac markers reflect gut damage with potential cardiac involvement, or the other way around with congestion casing intestinal oedema, cannot be answered in an observational study. Long‐term follow‐up with cardiac imaging in combination with microbiota analyses from the gut compartment would be the necessary next step to further test the potential impact of the gut‐heart axis in COVID‐19 patients.

Our study has several limitations. First, this study included a small number of patients and the results should be interpreted as exploratory. Second, we did not have plasma samples available for all patients at all time points, although the chosen statistical model does not require complete data set. Third, controls were not matched for age and gender, and comparisons between COVID‐19 patients and controls should be interpreted with caution. Furthermore, the diagnosis of cardiac involvement is based purely on elevated levels of cardiac markers as cardiac imaging were not feasible and does not allow us to separate between different etiologies of cardiac involvement including MI, myocarditis, heart failure or pulmonary hypertension with cardiac involvement.

In summary, our data show that hospitalized COVID‐19 patients with cardiac involvement were characterized by elevated markers of gut leakage and inflammasome activation. In light of our findings, COVID‐19 patients with cardiac involvement could be of particular relevance for intervention trials targeting inflammasome activation.

## Conflict of interest statement

The authors declare no conflict of interest.

## Funding

This study received funding from the Research Council of Norway grant no 312780 and has received private donation from Vivaldi Invest A/S owned by Jon Stephenson von Tetzchner.

## Author Contribution

**Hedda Hoel:** Conceptualization (equal); Formal analysis (equal); Writing‐original draft (equal). **Lars Heggelund:** Data curation (equal); Investigation (equal); Writing‐review & editing (equal). **Dag Henrik Reikvam:** Conceptualization (equal); Writing‐review & editing (equal). **Birgitte Stiksrud:** Conceptualization (equal); Writing‐review & editing (equal). **Thor Ueland:** Formal analysis (equal); Methodology (equal); Software (equal). **Annika Michelsen:** Methodology (equal); Writing‐review & editing (equal). **Kari Otterdal:** Writing‐review & editing (equal). **Karl Erik Muller:** Data curation (equal); Writing‐review & editing (equal). **Andreas Lind:** Data curation (equal); Writing‐review & editing (equal). **Fredrik Muller:** Project administration (equal); Writing‐review & editing (equal). **Susanne Dudman:** Data curation (equal); Writing‐review & editing (equal). **Pål Aukrust:** Conceptualization (equal); Writing‐review & editing (equal). **Anne Ma Dyrhol Riise:** Data curation (equal); Project administration (equal); Writing‐review & editing (equal). **Jan Cato Holter:** Data curation (equal); Project administration (equal); Writing‐review & editing (equal). **Marius Trøseid:** Conceptualization (lead); Supervision (lead); Writing‐original draft (equal).
